# Genetic variability in the precore and core promoter regions of hepatitis B virus strains in Karachi

**DOI:** 10.1186/1471-230X-6-20

**Published:** 2006-07-24

**Authors:** Zaigham Abbas, Rana Muzaffar, Arif Siddiqui, Syed Ali Anwar Naqvi, Syed Adibul Hassan Rizvi

**Affiliations:** 1 Department of Hepatogastroenterology, Sindh Institute of Urology and Transplantation, Karachi, Pakistan; 2 Department of Molecular Biology, Sindh Institute of Urology and Transplantation, Karachi, Pakistan

## Abstract

**Background:**

Hepatitis B virus (HBV) genotypes have distinct geographic distribution. Moreover, much genetic variability has been described in the precore (PC) and basal core promoter (BCP) regions of the HBV genome. The local prevalence of HBV genotypes and mutations has not been well studied. The aim of the present study is to determine the prevalence of HBV genotypes and mutations in the PC and BCP region in HBV strains in Karachi.

**Methods:**

A total of 109 chronic hepatitis B patients with detectable HBV DNA by a PCR assay were enrolled in the study. Sera were tested for HBeAg, anti-HBe antibody and liver profile. HBV genotypes and mutations in the PC and BCP regions were detected by INNO-LiPA line-probe assays.

**Results:**

Of the 109 patients investigated, 38 (35%) were HBeAg positive while 71 (65%) were HBeAg negative. Genotype D was present in 100% of the patients. Two patients had co-infection with genotype A. There was no significant difference in the baseline characteristics, mean ALT levels, and presence of clinical cirrhosis in patients with HBeAg positive or negative strains with or without PC and BCP mutations. Of the 38 HBeAg positive patients, 9 (24%) had PC and BCP mutations. In the HBeAg negative patient group, mutations were detected in 44 (62%) of the strains investigated. More than one mutation was common, seen in 26 (37%) patients with HBeAg negative disease and 6 (16%) patients with HBeAg positive disease. Twelve (17%) HBeAg negative patients had dual T1762 and A1764 mutations. None of the HBeAg positive patients had T1762 mutation. Mutations were undetectable in 27 (38%) of patients with HBeAg negative disease.

**Conclusion:**

Our study shows that type D is the main HBV genotype in Karachi, Pakistan. Significant numbers of patients infected with this genotype have PC and BCP variants. Mutations at more than one site are common. Patients harboring these mutants do not differ significantly in their clinical presentation from patients having wild type infection.

## Background

Hepatitis B Virus (HBV) is a well-known agent of acute and chronic hepatitis, liver cirrhosis and hepatocellular carcinoma. Around 400 million people worldwide carry the virus of which more than 250 million reside in Asia [1].

HBV has been classified into eight genotypes (A-H) according to the criterion of = 8% differences in the complete nucleotide sequence of the viral genome [2-5]. These genotypes show variation in their geographic distribution [4,6-8]. In addition to the epidemiological importance, these genotypes may influence the disease pattern and response to treatment.

In the natural course of chronic HBV infection, the loss of HBeAg expression and the appearance of antibodies directed against it (Anti-HBe) are usually accompanied by cessation of viral replication. However such a serology profile may also be seen in individuals who harbor precore (PC) and basal core promoter (BCP) mutants where replicative infection continues. The frequent genomic mutation that leads to HBeAg negativity is the mutation of the nucleotide (nt) 1896 from G to A (G-A). This mutation converts codon 28 of the precore sequence to a termination codon (TGG→TAG) and thus prevents HBeAg from being expressed [9]. PC variants are more common among patients with genotype D (65 to 75 percent) than genotype A (9 to 18 percent) [10,11].

A second group of mutations affect the basal core promoter region and result in a transcriptional reduction of precore but not pregenomic and core mRNA [12]. These HBeAg suppressive strains contain mutations of nt1762 from A to T (A 1762 T) and nt 1764 from G to A (G 1764 A) in the BCP region and are the predominant quasispecies in chronic hepatitis patients [13-16]. These mutations may be found in isolation or in conjunction with PC mutations. Occurrence of these mutations result in increase in viral load [12,13,17,18]. These changes were initially thought to be related to a "HBeAg-negative phenotype" but recent studies showed that they may also be found in some HBeAg-positive patients, especially those with chronic hepatitis [14,19].

The present study aimed to determine the HBV genotypes in Karachi and the pattern of PC and BCP mutations. This is the first study reporting the details of the PC and BCP mutants from Pakistan.

## Methods

Included in this study were 109 patients who visited the liver clinic of our institute for further evaluation and fulfilled the following criteria (1) HBsAg positive for more than six months (2) HBV DNA positive (3)No other concomitant liver diseases including hepatitis C and HIV infections, autoimmune hepatitis, Wilson's disease, primary biliary cirrhosis, alcoholic liver disease and non-alcoholic fatty liver disease (4) No previous treatment for hepatitis B. Demographic and clinical data of these patients was recorded. Ethics Review Committee of the hospital (SIUT) approved the protocol (see Additional file 1).

Informed consent was taken from patients fulfilling the inclusion criteria and blood samples taken. They were tested for alanine aminotransferase (ALT), alkaline phosphatase (ALP), serum albumin, bilirubin. Hepatitis B serological markers were tested by micro-ELISA method (AxSYM, Abbott laboratories, North Chicago, IL USA). HBV DNA was extracted from 200 μl of serum using QIAamp DNA Blood Mini Kit (Qiagen GmbH, Germany) and amplified by an in-house PCR assay with a detection limit of 1000 geq./ml. The primers used were P7 (5'-GTG GTG GAC TTC TCT CAA TTT TC-3' nt. 256–278) and S1–2 (5'-CGA ACC ACT GAA CAA ATG GC-3' nt. 685–704 [20].

HBV DNA positive samples were analyzed for viral genotypes and mutations in the PC and BCP regions. We used commercially available Line Probe Assays for HBV genotyping and mutation analysis (INNOGENETICS, Belgium) [11,21,22]. INNO-LiPA PreCore kit detects mutations in PC codon 28, and BCP nt 1762 and nt 1764. The kit can detect a wild type/mutant mixed population of circulating virus in contrast to sequencing which would only identify the predominant variant.

The patients were labeled suffering from clinical cirrhosis on the basis of clinical and ultrasound findings suggestive of cirrhosis (nodular surface, firm consistency, blunt liver edge, altered echotexure, dilated portal vein and splenomegaly) and evidence of hypersplenism (platelets < 100,00/mm^3^).

### Statistical analysis

The corrected chi-square test was used to compare categorical data. Student *t *test or one-way analysis of variance was used for group comparisons of parametric quantitative data and the Mann-Whitney test for similar comparisons of nonparametric data. In all cases, tests of significance were two-tailed, with a level at less than 0.05. Results are presented as median (range) or mean ± SD, whenever appropriate. Statistical analysis was done with the statistical package SPSS for Windows 2000 (version 10.1, SPSS, Chicago, IL).

## Results

Our study population included 109 HBV DNA positive chronic hepatitis B patients. The mean age of these patients was 32.6 ± 11.9 years (range 7–70 years). Eighty five (78%) patients were male. Thirty eight patients (35%) were HBeAg positive and 71 (65%) HBeAg negative. All the HbeAg negative patients and one HBeAg positive patients had Anti-HBe antibody positive. All of the samples belonged to genotype D except two patients who were coinfected with genotype A. Characteristics of these patients are given in Table 1. There was no difference in terms of sex, age, biochemical profile and clinical cirrhosis in HBeAg positive and negative variants.

Mutations in the PC and BCP regions were detected in 9 (24%) of HBeAg positive patients and 44 (62%) of HBeAg negative patients (p = 000). Remaining patients had the wild type band for both PC and BCP regions in the line probe assay. BCP mutations were found in 45 (41%) patients; 8 (21%) in HbeAg positive and 37 (52%) in HBeAg negative group. PC mutations were found in 7 (18%) and 22 (31%) of HbeAg positive and negative patients. Details of individual mutations are given in Table 2. More than one mutation was common, seen in 26 (37%) patients with HBeAg negative disease and 6 (16%) patients with HBeAg positive disease. Twelve (17%) HBeAg negative patients had dual T1762 and A1764 mutations. None of the HBeAg positive patients had T1762 mutation. Mutations were undetectable in 27 (38%) of patients with HBeAg negative disease.

The patients with wild type virus were younger than the patients having mutant type virus in HBeAg negative group (age 27.8 ± 9.6 vs 38.3 ± 12.9, *p *= 0.012). There were no statistical differences in the laboratory parameters and presence of clinical cirrhosis in patients harboring the wild virus compared to the mutant variant in both HBeAg negative and positive groups. Patients with normal ALT at the time of presentation did not differ from patients with elevated ALT in the frequency of different mutations.

## Discussion

All of our patients had genotype D with two patients coinfected with type A. This pattern is unique when compared to those of other countries in Asia. In South East Asia, genotypes B and C are highly prevalent [23] and in India a mixed pattern of genotypes D, A and C is seen [24,25]. There are therapeutic implications of this finding as patients with genotype D have more severe disease [26] and are less responsive to interferon therapy as compared to genotype A and B [27,28] and have higher HBV DNA levels (8). Moreover, in this genotype, specific viral sequence patterns present before initiation of therapy appear to be predictive of long-term response to lamivudine treatment [29].

The incidence of HBeAg negative chronic hepatitis B has increased in many countries [30]. In our study HBeAg negative patients predominated. This does not represent the true prevalence of the HBeAg negative disease as the samples were collected by convenience sampling. However, it does signify its high prevalence. Information about prevalence of PC and BCP mutations is also important in terms of response to interferon, development of fulminant hepatitis, rate of lamivudine resistant mutants and development of hepatocellular carcinoma [31]. Studies have shown that the PC variant is more prevalent among patients with genotype D, the type found in our patients, than in type A [10,11]. This mutation (G1896A) stabilizes the epsilon structure in the PC gene by forming a base pair with nt 1858 which exists as T in this genotype [32]. Treating these patients with interferon results in high relapse rates [33,34].

62% of our HBeAg negative patients and 24% of HBeAg positive patients had PC and BCP mutants (p = 000) so the frequency was more in those who did not express HBeAg. Combining HBeAg positive and negative groups PC variants were present in 29 (27%) and BCP variants in 44 (40%) patients. There was an overlap with mutations present at more than one site. There are some reports of association of PC mutation with fulminant hepatic failure [35] and the BCP variants with more severe liver damage [36-39] and HCC [40-43]. Other workers could not confirm a specific mutation predicting fulminant disease [44]. BCP mutations may not be necessarily pathogenic as they have been found in acute self limiting hepatitis, chronic hepatitis and asymptomatic carriers [45]. In our study, ALT levels and prevalence of clinical cirrhosis did not differ significantly in patients without or with PC or BCP mutants in both HBeAg negative and positive subgroups. Clinical relevance of these variants may be overstated and any correlation of nucleotide sequences with clinical and serologic findings must be done with precaution [46].

Studies from Asia suggested that the dual core promoter variant (A1762T, G1764A) was more common in patients with genotype C than those with genotype B [37,47]. Double mutation may enhance viral replication [13]. In our study most of the patients had HBV mutations at more than one sites. Twelve (17%) HBeAg negative patients had dual T1762 and A1764 mutations. None of the HBeAg positive patients had T1762 mutation.

In some of our patients though the line probe assay could not detect a specific mutation at 1762, 1764, and 1896 position but the wild PC or BCP band was absent. The samples were retested and the control bands worked in each run. This raises the possibility of other mutations in these regions undetected by INNO-LiPA (Table 2). It would be worthwhile investigating further these patients as it has been shown that three other nucleotide mutations at position 1817, 1874, 1897 also cause truncation in HBeAg and other changes that affect initiation codon at 1814, 1815 and 1816 have also been reported [48]. Moreover certain deletion mutations will not be detected by INNO-LiPA. Bozdayi et al [49] showed the existence of considerable variability between nucleotides 1751–1775 of the core promoter region in genotype D. Twenty seven (38%) of our HBeAg negative patients did not show any mutation by the assay we used.

Twenty four percent of our HBeAg positive patients had PC and BCP mutations. HBeAg did not disappear even in the presence of PC mutation. It may be due to presence of a mixed infection with the mutant and wild type viruses. Presence of wild type virus is needed by some of the mutants such as deletion mutants within the core promoter and core gene as well as variants with large deletions for infection of hepatocytes as shown for the woodchuck hepatitis virus [50]. It appears that HBV exists as a quasi species of wild type and mutant clones even in the HBeAg positive phase [46]. Direct sequencing can not detect a mutant population unless it accounts for a majority of the total population while INNO-LiPA assay is able to pick up the mixed infection.

Davidson F, et al compared the line probe assay we used with direct sequence analysis and found it more sensitive in detecting mixed infection. PC and BCP mutants were detected in three-fourth (74%) of patients with genotype D [51]. These mutations were picked up in half (49%) of cases in our study. In a study done by sequence analysis of Turkish patients with genotype D, BCP mutations were found in 17% and 29% of HbeAg positive and negative patient [49]. Our data suggests the figure of 21% and 52%. Perhaps geographical differences, host factors and type of assay used may influence the outcome.

## Conclusion

In conclusion, genotype D is the main HBV genotype in Karachi, Pakistan and significant number of our patients have PC and BCP variants. Patients harboring these mutants do not differ significantly in their clinical presentation from patients having wild type infection.

## Competing interests

The author(s) declare that they have no competing interests.

## Authors' contributions

ZA conceived of the study, wrote the protocol, coordinated different activities, did the statistical analysis and wrote the manuscript. RM performed assays and molecular studies, and helped in writing the manuscript and its revision. AS performed the data collection. SAAN helped with study design, contributed patients and revised manuscript. SAHR helped with the draft of manus and contributed patients. All authors read and approved the final manuscript.

## Pre-publication history

The pre-publication history for this paper can be accessed here:



## Figures and Tables

**Table 1:** Characteristics of patients, HBV genotypes and mutant

	**HBeAg Negative Patients **(n = 71)	**HBeAg Positive Patients **(n = 38)	***p *value**
Sex (M/F)	57/14	28/10	0.428
Age (Yrs)	33.8 ± 12.6	31.4 ± 11.5	0.464
Serum bilirubin (mg/dl)	1.4 ± 2.9	0.9 ± 0.8	0.247
ALT (U/L)	61 ± 79	93 ± 137	0.236
Alkaline phosphatase (U/L)	169 ± 124	195 ± 169	0.450
Serum Albumin (g/dl)	3.5 ± 1.0	3.6 ± 1.1	0.714
Platelet count (10^6^/μL)	179 ± 76	171 ± 80	0.751
Clinical cirrhosis	16	06	0.717
Genotype D*	71	38	1.000
Coinfection with genotype	00	02	
Mutations			
Precore G1896A	22	07	0.157
BCP A1762T	11	00	0.010^√^
BCP G1764A	27	06	0.016^√^
Absent wild PC band	22	05	0.040^√^
Absent wild BCP band	34	07	0.002^√^
Overall mutants**	44	09	0.000^√^
More than one mutations	26	06	0.023^√^

*Genotype D present in all the patients. **More than one mutations in many patients. PC: precore, BCP: basal core promoter. ^√^Significant *p *values

**Table 2:** Details of precore (PC) and basal core promotor (BCP) mutations as detected by line probe assays for PC 1896, BCP 1762, BCP 1764 and wild PC and BCP regions (n = 53)

**HBeAg Negative Patients (n = 44)**	**n**
PC (1896) alone	03
BCP 1762 alone	01
BCP 1764 alone	08
PC + 1764	06
BCP 1762 + 1764	03
PC + 1762 + 1764	06
PC + presence of wild PC band	02
PC + BCP 1762 + BCP 1764 + presence of wild PC band	02
Absent wild BCP band alone*	04
Absent PC wild band*	02
PC + absent wild BCP band	02
Miscellaneous combinations	05
**HBeAg Positive Patients (n = 9)**	
BCP 1764 + PC	04
BCP 1764 alone	01
Absent wild BCP band alone*	01
Miscellaneous combinations	03

PC = precore, BCP = basal core promoter

*Wild band and specified mutation bands absent. Thus mutations not detected by LiPA
